# Genetic polymorphisms in the endothelial nitric oxide synthase gene correlate with overall survival in advanced non-small-cell lung cancer patients treated with platinum-based doublet chemotherapy

**DOI:** 10.1186/1471-2350-11-167

**Published:** 2010-11-30

**Authors:** Shiro Fujita, Katsuhiro Masago, Yukimasa Hatachi, Akiko Fukuhara, Akito Hata, Reiko Kaji, Young Hak Kim, Tadashi Mio, Michiaki Mishima, Nobuyuki Katakami

**Affiliations:** 1 Division of Integrated Oncology, Institute of Biomedical Research and Innovation, 2-2 Minatojima Minami-machi, Chuo-ku, Kobe, Japan; 2 Department of Respiratory medicine, Graduate school of Medicine, Kyoto University, 54 Syogoin Kawaracho, Sakyo-ku, Kyoto, Japan

## Abstract

**Background:**

Nitric oxide (NO) is a free radical that is involved in carcinogenesis. Endothelial NO, synthesized from L-arginine by endothelial NO synthase (eNOS), inhibits apoptosis and promotes angiogenesis, tumor cell proliferation and metastasis. The aim of this study was to evaluate the influence of polymorphisms in the eNOS gene on prognosis of patients with advanced stage non-small-cell lung cancer (NSCLC).

**Methods:**

Unresectable, chemotherapy naïve stage III or IV NSCLC patients who were treated with standard platinum-containing doublet regimens were analyzed. All individuals were genotyped for the single-nucleotide polymorphism G894T in exon 7 of the eNOS gene and for a variable number of tandem repeats (VNTR) polymorphism in intron 4 that results in a rare smaller allele (a) and a common larger allele (b), to investigate the association between these polymorphisms and clinical outcomes. The primary endpoint was correlation with overall survival.

**Results:**

From October 2004 to December 2007, 108 patients (male/female, 66/42; Stage IIIA/IIIB/IV, 6/30/72) aged 29-77 years (median 63) with good performance status were consecutively enrolled in this study. Using Kaplan-Meier estimates, we showed that 5-year overall survival was significantly increased in patients carrying the VNTR a-allele compared with VNTR b/b patients (P = 0.015). In multivariate Cox proportional hazard analysis, the VNTR polymorphism was an independent prognostic factor for survival.

**Conclusions:**

The results support the role of the VNTR polymorphism in intron 4 as a marker for survival in patients with advanced stage NSCLC who are candidates for standard chemotherapy.

## Background

Lung cancer is the leading cause of cancer-related death in most developed countries [1]. Non-small-cell lung cancer (NSCLC) accounts for 80% of all lung cancers, and platinum-based chemotherapy is considered the standard of care worldwide for patients with advanced stage disease [2]. The treatment response is related to various factors, most of which are defined by tumor and clinical characteristics, such as disease stage and performance status. However, recent studies have shown that genetic factors may also influence the effectiveness of treatment [3,4].

Nitric oxide (NO) is a small free radical and is involved in numerous physiologic and pathophysiologic processes, including vasodilation, neurotransmission, immunity and carcinogenesis. NO is synthesized from L-arginine and oxygen by four major isoforms of NO synthase (NOS): neuronal NOS, endothelial NOS (eNOS), inducible NOS, and mitochondrial NOS. Although eNOS produces low levels of NO, many clinical observational studies have shown a dysregulation of eNOS expression in human solid tumors [5].

The number of reported polymorphisms in the gene encoding eNOS (*NOS3*) exceeds 160. The G894T polymorphism (rs1799983), which is located in exon 7 of the gene and leads to the amino acid change from Glu298Asp, is associated with reduced basal NO production [6]. In addition, a 27-bp variable number of tandem repeat (VNTR) polymorphism in intron 4 has been associated with variations in plasma levels of NO and its metabolites [7,8]. This polymorphism has three alleles: a short 4 repeat allele (a-allele), a more common long 5 repeat allele (b-allele), and a rare allele with 6 repeats. Recent studies have indicated that this repeat polymorphism in intron 4 is the source of a 27 nt-long RNA derived from pre-mRNA splicing. This RNA species inhibits eNOS expression and may represent a new class of small RNA [9,10]. Endothelial cells containing the a-allele produce a lower level of 27-nt small RNA and a higher level of eNOS mRNA compared with cells with the more common b-allele [10]. The association between eNOS mRNA levels and the a-allele appeared to be dose-dependent [11].

To date, very few studies have investigated the association of these polymorphisms with the prognosis of various neoplastic disorders, and no studies have examined them in relation to survival of advanced stage NSCLC patients who are candidates for standard platinum-based chemotherapy. In this study, we evaluated the potential association between these polymorphisms and survival of NSCLC patients.

## Methods

One hundred and eight patients who were judged to have inoperable stage III or IV NSCLC at the time of diagnosis were consecutively enrolled in our study between October 2004 and December 2007. All patients were native-Japanese of Asian ethnicity. Staging was based upon the 6th tumor node metastasis (TNM) staging system [12]. Patients with symptomatic brain metastasis, malignancies other than NSCLC within the last 5 years, presence of acute or chronic infections rendering them unsuitable for chemotherapy, or an Eastern Cooperative Oncology Group (ECOG) performance status (PS) of between 2 and 4 were excluded from the study. Patients with operable disease were also excluded, because surgery was considered to be a substantial confounding factor that masks the genetic effect on survival. Tumor response was evaluated according to the Response Evaluation Criteria in Solid Tumors (RECIST) [13], and all responses were evaluated at least 4 weeks after initial assessment. The local ethical committee (Kyoto University Graduate School and Faculty of Medicine, Ethics Committee) approved the study, and written informed consent was obtained from each patient before enrollment. The study was conducted in accordance with the Helsinki declaration.

The patients received one of the following three chemotherapy treatments: carboplatin/paclitaxel, carboplatin/gemcitabine or cisplatin/gemcitabine. With regard to the carboplatin/paclitaxel regimen, paclitaxel was administered either at a lower dose (70 mg/m2) on a weekly basis for 3 out of 4 weeks, or at 180 mg/m2 on day 1 (carboplatin dose was area under the curve (AUC) = 5 or 6 mg/mL • min on day 1). The combination of carboplatin and gemcitabine consisted of carboplatin AUC 5 mg/mL • min on day 1 plus gemcitabine 1000 mg/m2 on days 1 and 8 every 3 weeks. Carboplatin was replaced with cisplatin (80 mg/m2 on day 1) in the cisplatin/gemcitabine regimen. Each treatment was repeated for three to six cycles, unless unacceptable toxicity or disease progression was apparent. Patients who are candidate of definite thoracic irradiation therapy received carboplatin AUC 5 mg/mL • min on day 1 plus paclitaxel 40 mg/m2 on days 1, 8 and 15 every 4 weeks in a concurrent fashion. Two to four cycles of carboplatin/paclitaxel were administered subsequently for consolidation.

Blood samples were collected and DNA was isolated from whole blood cell fractions using the QIAamp DNA Blood Mini Kit (Qiagen, Tokyo, Japan). The single nucleotide polymorphism (SNP) of G894T (a G-to-T transversion at nucleotide 894 in exon 7) was analyzed with TaqMan genotyping assays, using the ABI 7500 real-time PCR system (Applied Biosystems, Inc., Tokyo, Japan). Probes, primers and TaqMan universal PCR master mix were those from ABI and TaqMan assays were performed according to the manufacturer's instructions. The intronic 27 bp insertion/deletion VNTR was genotyped using PCR amplification and agarose gel electrophoresis with ethidium bromide staining [14].

The distribution of genotypes was tested for Hardy-Weinburg equilibrium with the goodness-of-fit χ^2 ^test. The linkage disequilibrium of the two polymorphisms was analyzed. The association between overall survival times was estimated using the Kaplan-Meier method and assessed using the log-rank test. The effects of different genotypes on overall survival were estimated using hazard ratios (HRs), and 95% confidence intervals (95%CIs) were estimated using the multivariate Cox proportional hazards regression model, with adjustment for gender, age (<70 vs. older), ECOG PS (0 vs. 1), smoking status (never-smoker vs. former/current smoker), clinical stage (III vs. IV), histology (adenocarcinoma vs. others), thoracic radiotherapy (yes vs. no) and the two genotypes examined. We used Akaike's information criterion for entering and removing a variable in a stepwise Cox regression [15]. Survival time was calculated from diagnosis to death or last follow-up. A P value of <0.05 was considered statistically significant.

We estimated the false-positive report probability for the observed statistically significant associations using the methods described by Wacholder et al [16]. HR values from 2.0 to 4.0 were considered likely threshold values. The prior probability used was 0.1 for these polymorphisms and the false-positive report probability value for noteworthiness was set at 0.5.

Statistical analyses were performed using JMP version 6 (SAS Institute, Cary, NC, USA.), R software (R Foundation for Statistical Computing., Vienna, Austria.), and Haploview version 4.0 [17].

## Results

Patients' characteristics are summarized in Table 1. Eighty percent of patients had a cytological or histological diagnosis of adenocarcinoma. Treatment characteristics are shown in Table 2. By July 31, 2009, 73 (68%) of the 108 patients had died. The median follow-up period was 21.5 months.

**Table 1:** Characteristics of the 108 patients with non-small-cell lung cancer enrolled in the study

	Patients	
	
	N	%
Age, Years		
Median	63	
Range	29 - 77	
Gender		
Male	66	61.1
Female	42	38.9
Stage		
IIIA	6	5.5
IIIB	30	27.8
IV	72	66.7
PS		
0	23	21.3
1	85	78.7
Chemotherapy regimen		
CBDCA+PAC	89	82.4
CBDCA+GEM	17	15.7
CDDP+GEM	2	1.9
Definitive thoracic radiotherapy		
Yes	20	18.5
No	88	81.5

Abbreviations: CBDCA, carboplatin; PAC, paclitaxel; CDDP, cisplatin; GEM, gemcitabine

**Table 2:** Treatment characteristics of the enrolled patients

	No. of patients		
	
	Stage IIIA	Stage IIIB	Stage IV
CBDCA + PAC + TRT	6	14	0
CBDCA + PAC	0	13	56
CBDCA + GEM	0	3	14
CDDP + GEM	0	0	2

Abbreviations: TRT, thoracic radiotherapy

Median survival for all 108 patients was 26.8 months (95%CI, range 21.9 - 30.7 months). Median survival was 23.3 months for male patients and 32.3 months for female patients (HR, 1.72; 95%CI, 1.04-2.85; *P *= 0.034). Median survival was 24.6 months for smokers and 35.2 months for never-smokers (HR, 1.69; 95%CI, 1.00-2.85; *P *= 0.048). There was substantial agreement between gender and smoking status (Cohen's kappa coefficient, 0.64). Age, ECOG PS, disease stage, histology, and thoracic radiotherapy were not significantly associated with overall survival in the univariate analysis. Regarding epidermal growth factor receptor (EGFR) gene mutations, tumor samples of 34 patients (31.5%) were analyzed and 21 were positive for mutations. Eleven tumors had an L858R mutation in exon 21 and nine contained in-frame nucleotide deletions (including E746-A750) in exon 19. A G719S mutation was found in one tumor. EGFR mutation status (positive vs. negative/unknown) was not prognostic in the univariate analysis.

The distribution of all genotypes and allelic frequencies is shown in Table 3. These frequencies were in agreement with previous reports, and with those expected according to the Hardy-Weinburg equilibrium model. Linkage disequilibrium between these polymorphisms was not observed. Since there was no TT homozygote carrier with respect to G894T, and analyses were performed between the GG homozygote group and the GT heterozygote group. In addition, because the frequency of intron 4 short repeat (a-allele) homozygotes was low (0.9%; n = 1), b/a heterozygotes and a/a homozygotes were combined into an a-allele carrier group for analyses.

**Table 3:** Genotypic and allelic frequencies of the G894T SNP in exon 7 and the VNTR in intron 4

	Genotypic frequencies (%)			Allelic frequencies (%)	
G894T	GluGlu	GluAsp	AspAsp	Glu	Asp
	84.3	15.7	0	92.1	7.9
VNTR in intron 4	bb	ba	aa	b	a
	80.6	18.5	0.9	89.8	10.2

**Figure 1:**
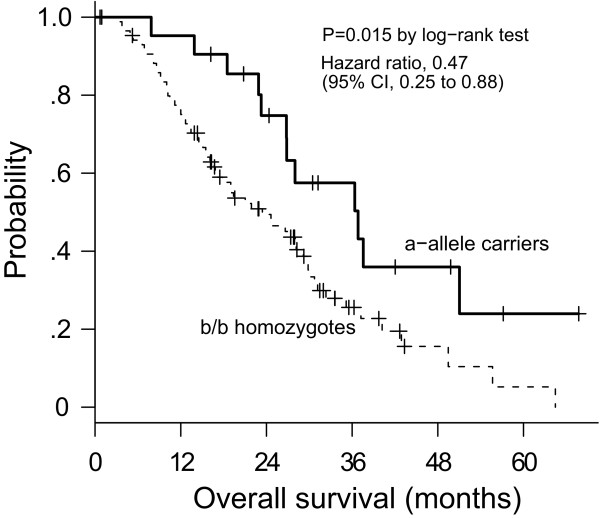
Kaplan-Meier plot of overall survival in relation to the VNTR genotype

**Figure 2:**
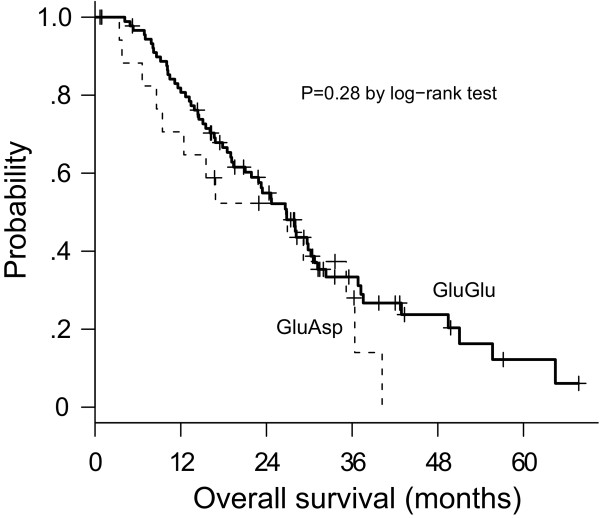
Kaplan-Meier plot of overall survival in relation to the G894T genotype in exon 7

Median survival was 36.8 months for a-allele carriers and 23.4 months for b/b homozygotes (Figure 1). The univariate Cox regression model showed that presence of the a-allele significantly correlated with prolonged survival (HR, 0.47; 95% CI, 0.25-0.88; *P *= 0.015). Correspondingly, carriers of the common b/b genotype had a higher risk of death than carriers of the a-allele (HR, 2.14). The false-positive report probability for the VNTR was 0.16, indicating noteworthiness. No significant difference in survival was observed according to the G894T SNP (*P *= 0.28) (Figure 2).

EGFR mutation status was not included in a Cox proportional hazards model because the number of analyzed patients was small and statistically significant results were not observed in the univariate analysis. Including gender, age, ECOG PS, smoking status, clinical stage, histology, thoracic radiotherapy and two genotypes in a model, the common b/b genotype (HR, 2.33; 95% CI, 1.24 - 4.36; *P *= 0.0085), male gender (HR, 2.02; 95% CI, 1.20-3.43; *P *= 0.0084) and stage IV disease (HR, 1.76; 95% CI, 1.04-2.99; *P *= 0.036) were selected as the predictors for overall survival. Correspondingly, HRs of female gender and stage III disease were 0.50 and 0.57, respectively.

With regard to clinical and pathological parameters, there were no discrepancies between carriers of the VNTR common b/b genotype and the VNTR a-allele carriers (Table 4). To investigate whether the eNOS intron 4 VNTR was associated with clinical benefit of chemotherapy, we analyzed the treatment response among patients without definitive thoracic radiotherapy. The overall response rate in 82 evaluable patients was 27%, with 22 (27%) partial responders (PR), 50 patients (61%) with stable disease (SD), and 10 patients (12%) with disease progression (PD). There was no significant distribution of a-allele carriers when patients were separated into two groups (PR+SD group vs. PD group; Table 5), indicating that the effect of the polymorphism on survival was not associated with the clinical benefit of first-line platinum-containing chemotherapy. We restricted the population to patients who received carboplatin plus paclitaxel without definitive thoracic radiotherapy and re-analyzed. The PR+SD rate was 81% in a-allele carriers and 86% in b/b genotype patients, respectively. Again there was no significant difference between the groups (*P *= 0.69).

**Table 4:** Comparative analysis of demographic and pathological information

	No. of bb genotype pts.	No. of a-allele carriers	P
Gender			
Female	35	7	P = 0.63
Male	52	14	
Age			
<70	64	15	P > 0.9
70 or older	23	6	
ECOG PS			
0	17	6	P = 0.38
1	70	15	
Smoking Status			
Never or Light	29	7	P > 0.9
Current or Former	58	14	
Clinical Stage			
III	31	5	P = 0.44
IV	56	16	
Histology			
Adenocarcinoma	68	18	P = 0.56
Non-adenocarcinoma	19	3	
EGFR mutation			
Activating mutation	19	2	P = 0.30
Wild type	9	4	
Unknown	59	15	
Thoracic radiotherapy			
Yes	17	3	P = 0.76
No	70	18	

**Table 5:** Frequency and distribution of eNOS intron 4 VNTR alleles in 72 patients with partial response or stable disease and 10 patients with progressive disease

	PR+SD		PD		
				
	No. of Patients	%	No. of Patients	%	P
a-allele carriers	15	83	3	17	0.68
bb genotype	57	89	7	11	

## Discussion

Our results show that polymorphism of the 27-bp VNTR in intron 4 of the eNOS gene is strongly associated with survival in advanced stage NSCLC patients treated with standard platinum-based chemotherapy. Median survival was significantly prolonged in patients carrying the shorter a-allele (i.e., those with a higher level of eNOS mRNA production).

Published studies indicate a procarcinogenic role of eNOS. In solid tumors, eNOS plays an essential role in VEGF-induced angiogenesis and vascular permeability [18]. A recent study showed that blocking phosphorylation of eNOS inhibited tumor initiation and maintenance through inactivation of the PI3K-AKT-eNOS-RAS pathway [19]. Moreover, a higher eNOS level was shown to correlate with cisplatin resistance in ovarian cancer cell lines [20]. Our findings were unexpected and in contrast to these observations.

However, our results can be explained by considering the following factors. NO derived from eNOS or eNOS itself can be cytotoxic to cancer cells through direct DNA damage and can be antitumorigenic [21]. The cytotoxic effects of NO can also be mediated through the generation of peroxynitrite (ONOO-) in the presence of superoxide anion (O∙2-) [22]. Moreover, clearance of metastatic cells from organ microvasculature is regulated in part by NO. NO has been reported to reduce the attachment of tumor cells to the endothelium and NO-mediated vasodilation may also contribute to the clearance of tumor cells [23,24]. Using an eNOS knockout mice model and the B16F1 melanoma cell line, Wang and colleagues have shown that arrest of tumor cell metastasis in the portal and pulmonary circulation can trigger the immediate release of NO in an eNOS-dependent manner, and subsequently induce apoptosis in these tumor cells [25,26]. Our observations were consistent with these reports. Furthermore, Mortensen et al. showed that expression of immunoreactive eNOS in the peritumoral microvasculature is a favorable prognostic indicator in premenopausal breast cancer patients, providing clinical evidence for a defensive role of eNOS against cancer cells [27]. With respect to treatment modality, NO was shown to increase the efficacy of chemotherapy and improve survival of patients with NSCLC in one phase II trial. Yasuda et al. randomized 120 patients with advanced stage NSCLC to receive standard chemotherapy (cisplatin and vinorelbine) alone, or the same regimen with nitroglycerin, a NO-donating drug [28]. The median time to progression was longer (10.9 vs. 6.2 months) in the nitroglycerin treatment group. Thus, in certain situations NO appears to act as a defense against the progression and/or metastasis of malignant tumors.

The median survival time of 26.8 months observed in the present study was favorable compared with that described in other reports [29,30]. This is probably due to the inclusion of a heterogeneous treatment group (including patients who received definitive chemoradiation) and the relatively young population (median 63 years of age).

Our study has several limitations. The single-institutional study design contains the potential to introduce a selection bias. Although we estimated the false-positive report probability to confirm the statistical significance obtained by established methods, analysis based upon a small number of enrolled patients could potentially lead to false-positive results. Two recent Japanese trials confirmed that EGFR mutation status is a predictive factor for response to EGFR-TKI treatment with a progression free survival of 10 months [31,32] and this finding paves the way for the personalized therapy for NSCLC. EGFR mutation status was evaluated in only a small proportion of the enrolled patients (31.5%) and the favorable effect of the mutations on prognosis was not observed in our study. Finally, because we restricted the study subjects to those of East-Asian ethnicity, it is uncertain whether these results can be generalized to other populations.

## Conclusions

Our results indicate that the VNTR polymorphism in intron 4 of the eNOS gene may be associated with the progression of NSCLC and shed light on the association between polymorphisms in the eNOS gene and survival of patients with NSCLC.

## Competing interests

The authors declare that they have no competing interests.

## Authors' contributions

SF participated in the design of the study, carried out the molecular genetic analysis and performed the statistical analysis. KM carried out the molecular genetic analysis, contributed to acquisition of data and helped to draft the manuscript. YH, AF, YHK and TM have made substantial contribution to acquisition of data. AH and RK performed the statistical analysis and helped to draft the manuscript. MM participated in the design of the study and helped to draft the manuscript. NK participated in the design of the study and conceived of the study. All authors read and approved the final manuscript.

## Pre-publication history

The pre-publication history for this paper can be accessed here:

http://www.biomedcentral.com/1471-2350/11/167/prepub

## References

[B1] Parkin DM Bray F Ferlay J Pisani P Global cancer statistics, 2002 CA Cancer J Clin 2005 55 74 108 10.3322/canjclin.55.2.74 15761078

[B2] Pfister DG Johnson DH Azzoli CG Sause W Smith TJ Baker S Jr Olak J Stover D Strawn JR Turrisi AT Somerfield MR American Society of Clinical Oncology treatment of unresectable non-small-cell lung cancer guideline: update 2003 J Clin Oncol 2004 22 330 353 10.1200/JCO.2004.09.053 14691125

[B3] de las Penas R Sanchez-Ronco M Alberola V Taron M Camps C Garcia-Carbonero R Massuti B Queralt C Botia M Garcia-Gomez R Isla D Cobo M Santarpia M Cecere F Mendez P Sanchez JJ Rosell R Polymorphisms in DNA repair genes modulate survival in cisplatin/gemcitabine-treated non-small-cell lung cancer patients Ann Oncol 2006 17 668 675 10.1093/annonc/mdj135 16407418

[B4] Rosell R Cecere F Santarpia M Reguart N Taron M Predicting the outcome of chemotherapy for lung cancer Curr Opin Pharmacol 2006 6 323 331 10.1016/j.coph.2006.01.011 16765644

[B5] Ying L Hofseth LJ An emerging role for endothelial nitric oxide synthase in chronic inflammation and cancer Cancer Res 2007 67 1407 1410 10.1158/0008-5472.CAN-06-2149 17308075

[B6] Veldman BA Spiering W Doevendans PA Vervoort G Kroon AA de Leeuw PW Smits P The Glu298Asp polymorphism of the NOS 3 gene as a determinant of the baseline production of nitric oxide J Hypertens 2002 20 2023 2027 10.1097/00004872-200210000-00022 12359981

[B7] Tsukada T Yokoyama K Arai T Takemoto F Hara S Yamada A Kawaguchi Y Hosoya T Igari J Evidence of association of the ecNOS gene polymorphism with plasma NO metabolite levels in humans Biochem Biophys Res Commun 1998 245 190 193 10.1006/bbrc.1998.8267 9535806

[B8] Wang XL Mahaney MC Sim AS Wang J Blangero J Almasy L Badenhop RB Wilcken DE Genetic contribution of the endothelial constitutive nitric oxide synthase gene to plasma nitric oxide levels Arterioscler Thromb Vasc Biol 1997 17 3147 3153 9409304 10.1161/01.atv.17.11.3147

[B9] Zhang MX Zhang C Shen YH Wang J Li XN Chen L Zhang Y Coselli JS Wang XL Effect of 27nt small RNA on endothelial nitric-oxide synthase expression Mol Biol Cell 2008 19 3997 4005 10.1091/mbc.E07-11-1186 18614799 PMC2526692

[B10] Zhang MX Zhang C Shen YH Wang J Li XN Zhang Y Coselli J Wang XL Biogenesis of short intronic repeat 27-nucleotide small RNA from endothelial nitric-oxide synthase gene J Biol Chem 2008 283 14685 14693 10.1074/jbc.M801933200 18390539 PMC2386909

[B11] Senthil D Raveendran M Shen YH Utama B Dudley D Wang J Wang XL Genotype-dependent expression of endothelial nitric oxide synthase (eNOS) and its regulatory proteins in cultured endothelial cells DNA Cell Biol 2005 24 218 224 10.1089/dna.2005.24.218 15812238 PMC1350115

[B12] Mountain CF Revisions in the International System for Staging Lung Cancer Chest 1997 111 1710 1717 10.1378/chest.111.6.1710 9187198

[B13] Therasse P Arbuck SG Eisenhauer EA Wanders J Kaplan RS Rubinstein L Verweij J Van Glabbeke M van Oosterom AT Christian MC Gwyther SG New guidelines to evaluate the response to treatment in solid tumors J Natl Cancer Inst 2000 92 European Organization for Research and Treatment of Cancer, National Cancer Institute of the United States, National Cancer Institute of Canada 205 216 10.1093/jnci/92.3.205 10655437

[B14] Miyahara K Kawamoto T Sase K Yui Y Toda K Yang LX Hattori R Aoyama T Yamamoto Y Doi Y Ogoshi S Hashimoto K Kawai C Sasayama S Shizuta Y Cloning and structural characterization of the human endothelial nitric-oxide-synthase gene Eur J Biochem 1994 223 719 726 10.1111/j.1432-1033.1994.tb19045.x 7519987

[B15] Akaike H Petrov BN, Czáki F Information Theory and Extension of the Maximam Likelifood Principle Second International Symposium on Information Theory 1973 Budapest: Akademiai Kiadó 267 281

[B16] Wacholder S Chanock S Garcia-Closas M El Ghormli L Rothman N Assessing the probability that a positive report is false: an approach for molecular epidemiology studies J Natl Cancer Inst 2004 96 434 442 10.1093/jnci/djh075 15026468 PMC7713993

[B17] Barrett JC Fry B Maller J Daly MJ Haploview: analysis and visualization of LD and haplotype maps Bioinformatics 2005 21 263 265 10.1093/bioinformatics/bth457 15297300

[B18] Duda DG Fukumura D Jain RK Role of eNOS in neovascularization: NO for endothelial progenitor cells Trends Mol Med 2004 10 143 145 10.1016/j.molmed.2004.02.001 15162796

[B19] Lim KH Ancrile BB Kashatus DF Counter CM Tumour maintenance is mediated by eNOS Nature 2008 452 646 649 10.1038/nature06778 18344980 PMC2688829

[B20] Leung EL Fraser M Fiscus RR Tsang BK Cisplatin alters nitric oxide synthase levels in human ovarian cancer cells: involvement in p53 regulation and cisplatin resistance Br J Cancer 2008 98 1803 1809 10.1038/sj.bjc.6604375 18506185 PMC2410127

[B21] Sonveaux P Jordan BF Gallez B Feron O Nitric oxide delivery to cancer: why and how? Eur J Cancer 2009 45 1352 1369 10.1016/j.ejca.2008.12.018 19153039

[B22] Reiter CD Teng RJ Beckman JS Superoxide reacts with nitric oxide to nitrate tyrosine at physiological pH via peroxynitrite J Biol Chem 2000 275 32460 32466 10.1074/jbc.M910433199 10906340

[B23] Kong L Dunn GD Keefer LK Korthuis RJ Nitric oxide reduces tumor cell adhesion to isolated rat postcapillary venules Clin Exp Metastasis 1996 14 335 343 10.1007/BF00123392 8878407

[B24] Yamamoto T Terada N Seiyama A Nishizawa Y Akedo H Kosaka H Increase in experimental pulmonary metastasis in mice by L-arginine under inhibition of nitric oxide production by NG-nitro-L-arginine methyl ester Int J Cancer 1998 75 140 144 10.1002/(SICI)1097-0215(19980105)75:1<140::AID-IJC21>3.0.CO;2-J 9426702

[B25] Qiu H Orr FW Jensen D Wang HH McIntosh AR Hasinoff BB Nance DM Pylypas S Qi K Song C Muschel RJ Al-Mehdi AB Arrest of B16 melanoma cells in the mouse pulmonary microcirculation induces endothelial nitric oxide synthase-dependent nitric oxide release that is cytotoxic to the tumor cells Am J Pathol 2003 162 403 412 12547699 10.1016/S0002-9440(10)63835-7 PMC1851169

[B26] Wang HH McIntosh AR Hasinoff BB Rector ES Ahmed N Nance DM Orr FW B16 melanoma cell arrest in the mouse liver induces nitric oxide release and sinusoidal cytotoxicity: a natural hepatic defense against metastasis Cancer Res 2000 60 5862 5869 11059784

[B27] Mortensen K Holck S Christensen IJ Skouv J Hougaard DM Blom J Larsson LI Endothelial cell nitric oxide synthase in peritumoral microvessels is a favorable prognostic indicator in premenopausal breast cancer patients Clin Cancer Res 1999 5 1093 1097 10353743

[B28] Yasuda H Yamaya M Nakayama K Sasaki T Ebihara S Kanda A Asada M Inoue D Suzuki T Okazaki T Takahashi H Yoshida M Kaneta T Ishizawa K Yamanda S Tomita N Yamasaki M Kikuchi A Kubo H Sasaki H Randomized phase II trial comparing nitroglycerin plus vinorelbine and cisplatin with vinorelbine and cisplatin alone in previously untreated stage IIIB/IV non-small-cell lung cancer J Clin Oncol 2006 24 688 694 10.1200/JCO.2005.04.0436 16446342

[B29] Sandler A Gray R Perry MC Brahmer J Schiller JH Dowlati A Lilenbaum R Johnson DH Paclitaxel-carboplatin alone or with bevacizumab for non-small-cell lung cancer N Engl J Med 2006 355 2542 2550 10.1056/NEJMoa061884 17167137

[B30] Scagliotti GV Parikh P von Pawel J Biesma B Vansteenkiste J Manegold C Serwatowski P Gatzemeier U Digumarti R Zukin M Lee JS Mellemgaard A Park K Patil S Rolski J Goksel T de Marinis F Simms L Sugarman KP Gandara D Phase III study comparing cisplatin plus gemcitabine with cisplatin plus pemetrexed in chemotherapy-naive patients with advanced-stage non-small-cell lung cancer J Clin Oncol 2008 26 3543 3551 10.1200/JCO.2007.15.0375 18506025

[B31] Maemondo M Inoue A Kobayashi K Sugawara S Oizumi S Isobe H Gemma A Harada M Yoshizawa H Kinoshita I Fujita Y Okinaga S Hirano H Yoshimori K Harada T Ogura T Ando M Miyazawa H Tanaka T Saijo Y Hagiwara K Morita S Nikiwa T Gefitinib or chemotherapy for non-small-cell lung cancer with mutated EGFR N Engl J Med 2010 362 2380 2388 10.1056/NEJMoa0909530 20573926

[B32] Mitsudomi T Morita S Yatabe Y Negoro S Okamoto I Tsurutani J Seto T Satouchi M Tada H Hirashima T Asami K Katakami N Takada M Yoshioka H Shibata K Kudoh S Shimizu E Saito H Toyooka S Nakagawa K Fukuoka M Gefitinib versus cisplatin plus docetaxel in patients with non-small-cell lung cancer harbouring mutations of the epidermal growth factor receptor (WJTOG3405): an open label, randomised phase 3 trial Lancet Oncol 2010 11 121 128 10.1016/S1470-2045(09)70364-X 20022809

